# Mechanistic and Therapeutic Implications of Protein and Lipid Sialylation in Human Diseases

**DOI:** 10.3390/ijms252211962

**Published:** 2024-11-07

**Authors:** Xiaotian Zhong, Aaron M. D’Antona, Jason C. Rouse

**Affiliations:** 1 BioMedicine Design, Discovery and Early Development, Pfizer Research and Development, 610 Main Street, Cambridge, MA 02139, USA; aaron.dantona@pfizer.com; 2 Analytical Research and Development, Biotherapeutics Pharmaceutical Sciences, Pfizer Inc., Andover, MA 01810, USA; jason.rouse@pfizer.com

**Keywords:** sialylation of glycoproteins and glycolipids, gangliosides, glycocalyx, Siglecs and Selectins, human diseases, clinical development of therapies

## Abstract

Glycan structures of glycoproteins and glycolipids on the surface glycocalyx and luminal sugar layers of intracellular membrane compartments in human cells constitute a key interface between intracellular biological processes and external environments. Sialic acids, a class of alpha-keto acid sugars with a nine-carbon backbone, are frequently found as the terminal residues of these glycoconjugates, forming the critical components of these sugar layers. Changes in the status and content of cellular sialic acids are closely linked to many human diseases such as cancer, cardiovascular, neurological, inflammatory, infectious, and lysosomal storage diseases. The molecular machineries responsible for the biosynthesis of the sialylated glycans, along with their biological interacting partners, are important therapeutic strategies and targets for drug development. The purpose of this article is to comprehensively review the recent literature and provide new scientific insights into the mechanisms and therapeutic implications of sialylation in glycoproteins and glycolipids across various human diseases. Recent advances in the clinical developments of sialic acid-related therapies are also summarized and discussed.

## 1. Introduction

The Swedish biochemist Gunnar Blix first coined the term “sialic acid” from the Greek word saliva for the acidic compounds originally isolated from salivary glands, now referred to a class of alpha-keto acid sugars with a nine-carbon backbone [1,2,3]. Independently, Ernst Klenk discovered neuraminic acids in brain glycosphingolipids (GSL), which were later shown to be identical to the sialic acids identified previously by Blix [2,4,5]. Both sialic acid (Sia) and neuraminic acid (Neu) terminologies have been used throughout the literature to describe the group of nine-carbon monosaccharides (5-amino, 3,5-dideoxy-*D*-glycero-*D*-galacto-nonulosonic acid) containing three functional groups: a carboxyl group at the C2 anomeric carbon, a glycerol-like three-carbon side chain at the C6 carbon, and an amino-acyl group or hydroxyl group attached to the C5 carbon [2,6,7]. Sia now refers to the entire family, whereas Neu refers to the C-5 free amine form. There are three main structures of sialic acids in nature: N-acetylneuraminic acid (Neu5Ac), N-glycolylneuraminic acid (Neu5Gc), and keto-deoxynonulosonic acid. These Sia molecules can be further modified with O-substitutions at C-4, C-7, C-8, and C-9 by acetylation, lactylation, methylation, phosphorylation, and sulfation, resulting in a family with over 50 different naturally occurring members. 

Different from the sugars with a six-carbon backbone, the Sia molecules are negatively charged and only found at the termini of glycan branches, except within other Sial molecules. The Sia molecules can be found in the *N*-linked (via Asn) and *O*-linked (via Ser/Thr) glycans of glycoproteins as well as in the sugar moiety of glycolipids (termed gangliosides) [8]. They can form a glycosidic linkage with themselves, *D*-galactose (Gal), *N*-acetylgalactosamine (GalNAc), and *N*-acetyl-*D*-glucosamine (GlcNAc). According to the anomeric carbon C1 of sialic acids, the glycosidic bonds are formed at the α position of C2 through the hydroxyl group at C3/C6 of Gal, at C6 of GalNAc, and at C4, C8, C9, and C5-O_glycolyl_ (Neu5Gc) of Sia. An additional level of structural diversity is the different degrees of polymerization of Sia residues, such as di (DP = 2), oligo (DP = 3–7), and poly (DP = 8–400) [9]. Sialic acids are plentiful on the carbohydrate-rich coating layers of animal cell surfaces, and Martinex-Palomo introduced the term “glycocalyx” [10] to describe this key interface for regulating diverse biological processes [11,12,13,14]. 

Like other sugar addition processes, adding sialic acid to glycan branches is a non-template process that is strictly regulated by the specificity and availability of enzymatic substrates as well as the protein subcellular localization. Mammalian sialyltransferases (STs) [7,15,16] that are responsible for this reaction belong to a large group of inverting glycosyltransferases regarded as the glycotransferase GT-29 family of the CAZy database (http://www.cazy.org/) [17]. These Golgi-localized membrane-bound enzymes catalyze the transfer of Sia from the nucleotide-activated sugar donor, e.g., cytidine 5′-monophosphate-β-Neu5Ac (CMP-Neu5Ac), to the non-reducing end of a growing carbohydrate chain linked to either a protein or a lipid, with a high reaction fidelity and a high acceptor specificity. In the reverse direction, the Sia molecules in the glycans can be removed by sialidases [18,19,20,21,22,23,24,25,26]. The cellular Sia content in the glycocalyx is controlled by the actions of both STs and sialidases.

With the polar and charge nature as well as the terminal location at the glycan chains of the glycocalyx, the Sia molecules play important functional roles in many biological processes of animal cells [2,5,7,13,14,27]. They can maintain cell shape, provide binding sites for molecules, cells, and pathogens, and are involved in cell recognition, migration, and metastasis. The sialylated bioconjugates in the glycans on the glycoproteins and those linked to the glycolipids are synthesized through distinct molecular pathways. A number of recent reviews have summarized the biological impacts of these glycans on human physiologies and diseases. Some of these review articles focus on the biosynthesis of *N*- or *O*-linked glycans [7,11,28,29,30,31,32,33,34,35,36,37,38,39,40,41,42,43,44], whereas some concentrate on polysialic acids [9,45,46] or gangliosides [7,13,31,47,48,49,50,51,52,53,54,55,56,57,58,59,60]. The roles of the glycans in health and diseases such as autoimmune diseases [14,26,28,36,60,61,62,63,64,65,66,67,68], cancers [13,14,16,30,31,33,36,38,39,40,41,47,49,55,57,58,64,67,68,69,70,71,72,73,74,75,76,77,78,79,80,81,82,83,84,85,86], cardiovascular diseases [25,42,87], fibrosis [88], neurodegenerative and psychiatric diseases [9,48,50,51,53,56,59,64,76,89,90,91,92,93,94], infectious diseases [20,26,66,95,96,97], free sialic acid storage disorders [34], or lysosomal storage diseases [51,54] have also been reviewed. Various therapeutic strategies, such as targeting Sia-binding protein Siglecs (Sialic acid-binding immunoglobulin-type lectins)/Selectins or gangliosides [11,14,35,47,52,62,63,64,65,66,67,68,70,74,75,76,77,82,83,84,85,86,89,91,92,94,95,96,98,99,100,101,102,103], have also been discussed. Recent developments in ST inhibitors as potential treatments based on the current knowledge of structure-function relationships and molecular implications in human biology are summarized [20,70,71,80,81]. The development of tools for altering the total composition of cellular sialic acids—the sialome [11,78,96,104]—and engineering intracellular glycan sialylation pathways for diverse protein, gene, and cell therapies has been highlighted [29,31,32,105,106,107,108]. 

This review article mainly focuses on the sialylation aspects of glycoproteins and glycolipids, which are the only known cellular macromolecules for sialylated glycans. The molecular machineries responsible for the biosynthesis and catabolism of sialylated glycans for glycoproteins and glycolipids in mammalian cells are comparatively summarized in parallel. Scientific insights into the therapeutic implications of sialylation in glycoproteins and glycolipids across various human diseases are provided through a comprehensive review of the recent literature. Therapeutic strategies targeting protein and lipid sialylation against cancer, cardiovascular, neurological, inflammatory, infectious, and lysosomal storage diseases are summarized. Recent advances in the clinical developments of sialic acid-related therapies are also reviewed and discussed.

## 2. Enzymatically Distinct and Functionally Similar Molecular Machineries Involved in the Sialyation of Glycoproteins and Glycolipids with Diverse Glycan Substrates

**Figure 1:**
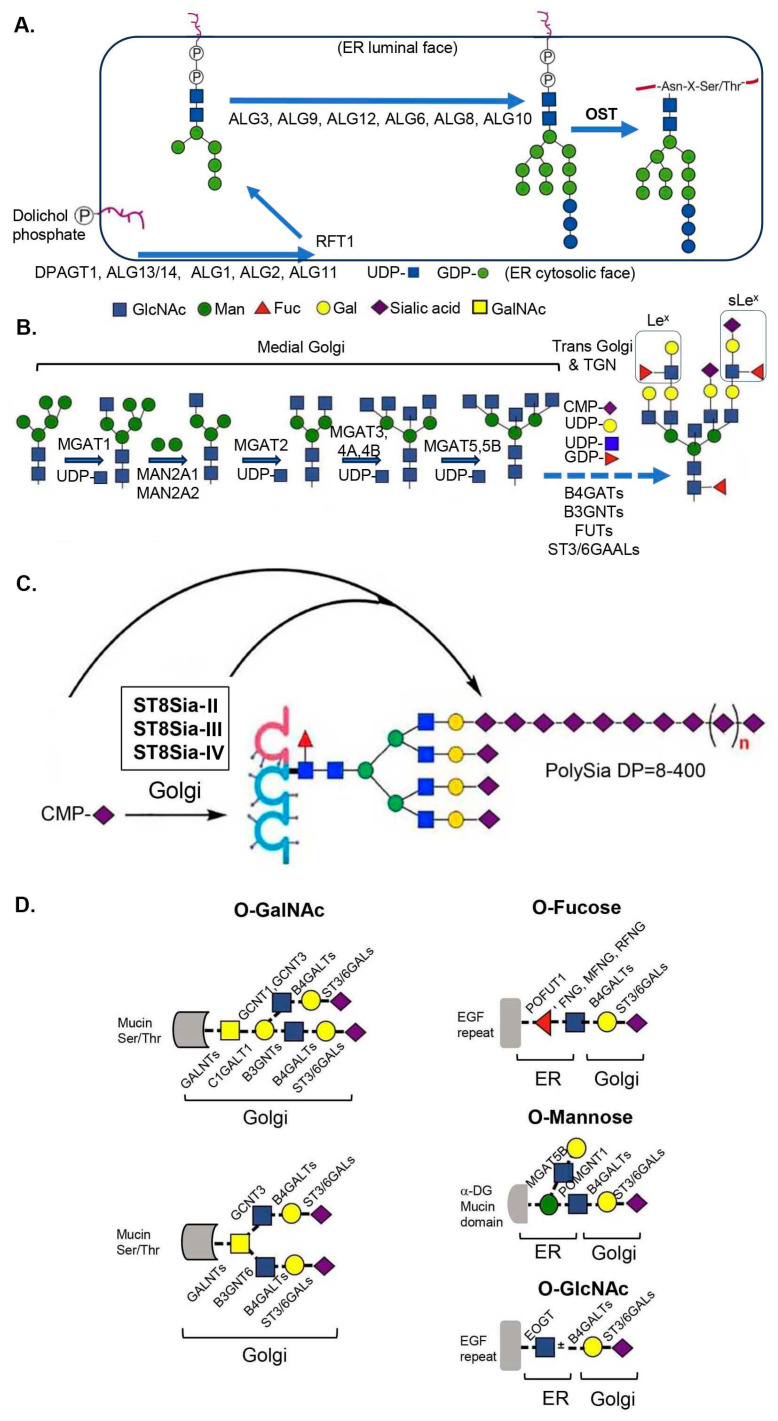
Sialylation pathways in glycoproteins. The protein motifs, sugar symbols, subcellular compartments, and glycosyltransferases involved are shown. (**A**) Core *N*-glycosylation in ER [8]. (**B**) *N*-glycan sialylation in the Golgi [8,109]. (**C**) Polysialylation in the Golgi [45]. (**D**) *O*-glycan sialylation pathways [8,109].

**Figure 2:**
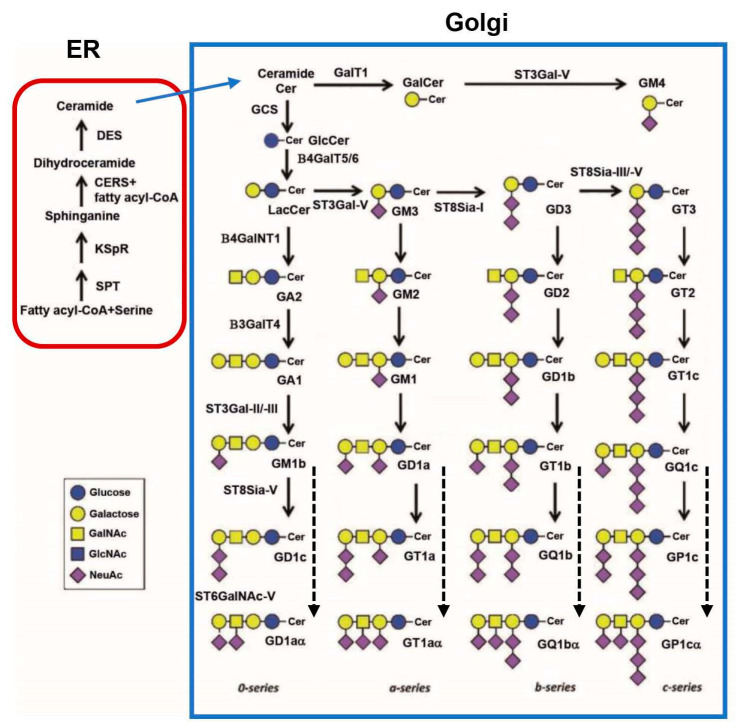
Sialylation pathways for glycosphingolipids [13,48,59]. Reaction steps and the enzymes involved in the biosynthetic pathways, sugar symbols, as well as subcellular compartments, and the glycosyltransferases involved are shown. According to the nomenclature [110], “G” states the ganglio-series of glycosphingolipids, whereas “A, M, D, T, Q, P” indicate the presence of zero (absent), one (mono-), two (di-), three (tri), four (quadra), and five (penta) sialic acid residues, respectively. Gangliosides with core structure “Galβ1-3GalNAcβ1-4Galβ1-4Glcβ1-1Cer” are indicated with number “1”, those lacking the terminal Gal are labeled with “2”, those lacking the terminal Galβ1-3GalNAc are labeled with “3”, and GM4 is a ganglioside of GalCer. The “*0-, a-, b-, c-*” series include gangliosides with 0, 1, 2, and 3 sialic acid residues linked to innermost Gal.

**Figure 3:**
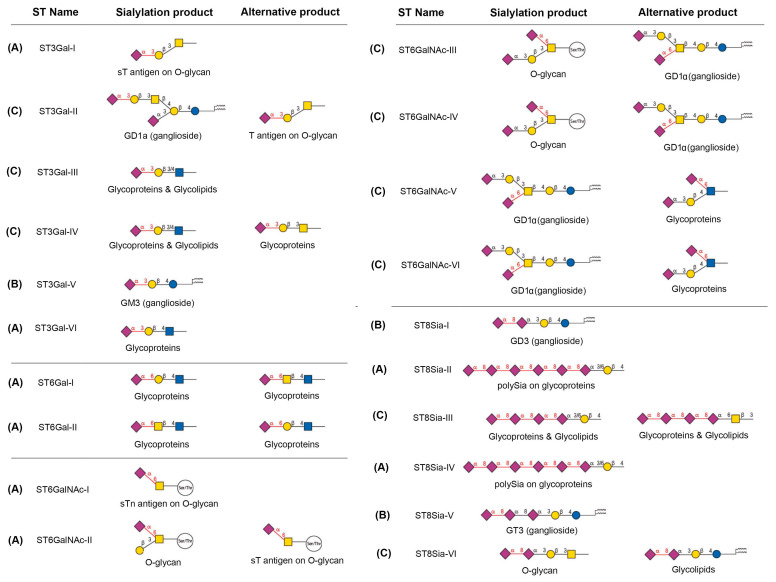
Enzymatic specificities for human STs [7]. Four groups and twenty members of human STs are listed. The preferred and alternative products they produce are indicated. Classification A–C based on substrate specificity toward glycoproteins or glycolipids are also designated.

Sialylated glycan chains in glycoproteins and glycolipids are de novo synthesized through distinct molecular pathways in mammalian cells (Figure 1 vs. Figure 2). The respective machineries share similarities, such as the involvements of cytoplasmic and lumen faces of the endoplasmic reticulum (ER) as well as the Golgi lumen, but there are a few major differences between the sialylated glycan chains in glycoproteins and glycolipids. Firstly, the compositions of glycan moieties in glycoproteins and glycolipids are different. Those in the glycoproteins are made of seven monosaccharides found in the sialylated glycans: Gal, *D*-glucose (Glc), *D*-mannose (Man), *L*-fucose (Fuc), GlcNAc, GalNAc, and Neu5Ac, along with those only found in non-sialylated glycans: *D*-xylose (Xyl), *D*-glucuronic acid, and iduronic acid. Those in the glycolipids are mainly made of six monosaccharides: Glc, Gal, GalNAc, GlcNAc, Fuc, and Neu5Ac. Secondly, the machinery distribution for the synthesis locations is different. The synthesis of the sialylated glycans for the glycoproteins takes place relatively equally in the ER and the Golgi, whereas those for the glycolipids almost exclusively occur in the Golgi. Thirdly, because of the more diverse glycan structures found in glycoproteins compared to those in glycolipids, substantially more glycogenes such as glycotransferases are involved in glycan biosynthesis for glycoproteins (Figure 1) than in the synthesis of glycolipids (Figure 2). Lastly, for sialylation modification, significantly more STs (Figure 3) are utilized for the modification of the glycoproteins’ glycans, and there are also sialylated glycans unique to glycoproteins, such as polysialyated glycans with a degree of polymerization (DP) ≥ 8.

For glycan chains in the glycoproteins, two independent sets of molecular machineries are involved in the synthesis of *N*-linked and *O*-linked glycans [111,112,113]. *N*-linked glycans start from core modification by the *en bloc* transfer via oligosaccharyltransferase (OST) with trimmings of Glc and Man in the ER. The core glycan Glc_3_Man_9_GlcNAc_2_ is pre-synthesized by a series of enzymatic processes initiated on the cytoplasmic face of the ER and then transited to the ER lumen (Figure 1A). Further *N*-glycan modifications such as GlcNAc branching, galactosylation, fucosylation, and sialylation during the complex *N*-glycan synthesis take place in the Golgi (Figure 1B). The sialylation patterns for *N*-glycans include α2,3 sialylation, α2,6 sialylation, and polysialylation (polySia) [9,45]. PolySia is highly regulated in mammals, which occurs thus far at the glycan modification for a restricted group of polySia protein carriers in the central nervous system (CNS) and the immune system [9,45]. ST8Sia-II, ST8Sia-III, and ST8Sia-IV were responsible for the polySia in the Golgi of different tissues [45,114] (Figure 1C). For the *O*-linked glycosylation, there are seven different types of first-linked sugars to Ser/Thr residues or hydroxylysine (HYL), including GalNAc-, Fuc-, GlcNAc-, Man-, Glc-, Xyl-, and HYL-Gal [109,111,112,113]. Glc- and HYL-Gal-types of *O*-linked glycosylation take place in the ER, which is not further modified with sialylation [109]. HYL-Gal *O*-glycosylation is typically limited to collagens and some therapeutic proteins expressed in CHO cells [115]. Mammalian Man-, GlcNAc-, and Fuc-types of *O*-linked glycosylations are also initiated in the ER and further modified in the Golgi, including sialylation (Figure 1D). GalNAc-type and Xyl-type *O*-linked glycosylation start at the Golgi by polypeptide GalNAc transferases (GALNTs) and O-xyltransferases (XYLTs), respectively. Xyl-type O-linked glycosylation is not modified by sialylation, whereas GalNAc mucin-type O-linked glycosylation with core-1-6 structures can be further modified with the α2,3 and α2,6 sialylations. Therefore, four types of *O*-linked glycans (GalNAc-, Fuc-, Man-, and GlcNAc-types) can carry terminal sialylated residues (Figure 1B). 

The biosynthesis of the sialylated glycolipid gangliosides (Figure 2) starts from the cytoplasmic membrane leaflet of the ER for the formation of ceramide anchor, as the synthesis of the core N-glycan (Figure 1A), followed by the glycan synthesis occurred exclusively in the Golgi [13,48,50,51]. By condensing activated fatty acids with L-serine through serine palmitoyl transferase (SPT) and reduction via ketodihydrophingosine reductase (KSpR), sphinganine/dihydrosphingosine is produced. Through one of six ceramide synthases (CERS) which have different specificities towards activated fatty acids of various chain lengths, sphinganine is converted to dihydroceramide. The sphingosine double bond is then introduced by a dihydroceramide desaturase (DES) to form ceramides. After exiting the ER, the primary hydroxyl group of ceramides can be modified with Gal to form GalCer by ceramide galactosyltransferase (CGT/GalT1). The attachment of glucose by glucosylceramide synthase (GCS) generates GlcCer, which is the structural base for the majority of GSLs and gangliosides. GalCer can be further modified with one Sia by ST3Gal-V to form GM4, whereas GlcCer is modified with one Gal by B4GalT5/6 to form Lactosylceramide (LacCer), the fundamental branching point in the GSL synthesis (Figure 2). From the horizontal branch, LacCer is modified with one Sia by ST3Gal-V to form GM3 which can be added with one more Sia by ST8Sia-I to generate GD3. ST8Sia-II/-V can add one additional Sia to form GT3. These products (GM3, GD3, and GT3) along with LacCer can be further modified via the vertical direction (Figure 2). For instance, B4GalNT1 can add one GalNAc to the Gal of LacCer to form GA2. B3GalT4 then adds one Gal to the GalNAc of GA2 to form GA1. ST3Gal-II/-III can add one Sia to the Gal of GA1 to form GM1b. ST8Sia-V can add one additional Sia to the Sia of GM1b to form GD1c. Alternatively, ST6GalNAc-V can add one Sia to the GalNAc of GM1b to form GD1aα to complete the *0*-series of the gangliosides. By this set of enzymes on the vertical branch (Figure 2), GM3 can form the *a*-series of the gangliosides (GM2, GM1, GD1a, GT1a, and GT1aα), GD3 for the *b*-series (GD2, GD1b, GT1b, GQ1b, and GQ1bα), and GT3 for the *c*-series (GT2, GT1c, GQ1c, GP1c, and GP1cα). In the adult brain, GM1, GD1a, GD1b, and GT1b are the major gangliosides, whereas GM3 predominates in most peripheral tissues.

For all the sialylation modifications (glycoproteins and glycolipids) in mammals, about 20 ST genes (Figure 3) are found in the human genome, with more than 155 ST genes identified in 25 animal species [15,116,117], recognizing diverse glycan substrates through the linkages with Gal, GalNAc/GlcNAc, or Sia residue itself. These genes are polyexonic in terms of genomic organization and widely dispersed on several different chromosomes. At least three pseudogenes have been reported in the human genome [7]. These STs, predominantly located in the trans-Golgi compartment, are type II transmembrane glycoproteins with a short N-terminal cytoplasmic tail, a unique transmembrane domain, and a stem region of variable length followed by the C-terminal enzymatic domain. Despite limited overall sequence identity, all the ST catalytic domains share four peptide-conserved motifs called the sialylmotifs: L (Large), S (small), motif III, and motif VS (very small). These hallmark sequences are implicated in the catalytic reaction as well as the recognition of both donor and acceptor substrates [7,117,118,119]. The roles of the L motif in the binding of the CMP-Sia donor, the S motif in interacting with both the CMP-Sia and the acceptor substrates, the VS motif in the catalytic reaction, and motif III in the acceptor recognition and the catalytic efficiency, have been established. Detailed enzymatic mechanisms for STs have been reviewed by two recent articles [7,117].

Based on the acceptor glycoconjugates terminating monosaccharides as well as the position of the attachment of the donor Sia to the acceptor, the ST family has been classified into four groups (Figure 3): (1) the ST3Gal family, transferring Neu5Ac residues in an α2,3-linkage to terminal Gal residues found in glycoproteins or glycolipids; (2) the ST6Gal family, which catalyzes the transfer of Neu5Ac in an α2,6-linkage to the hydroxyl group in C6 of terminal Gal in the Galβ1-4GlcNAc; (3) the ST6GalNAc family, which catalyzes the transfer of Neu5Ac residues in an α2–6 linkage to the GalNAc residues found in the *O*-glycosylproteins or the glycolipids; and (4) the ST8Sia family, which mediates the transfer of Neu5Ac residues in an α2,8-linkage to other Neu5Ac residues found in glycoproteins and glycolipids.

With regards to the enzymatic specificities towards the glycan substrates of glycoproteins and glycolipids [7,16], the STs can be further classified into three subgroups (Figure 3). Members of subgroup A are unique to sialylation in the glycoproteins. They are ST3Gal-I and -VI for *N*-/*O*-glycans, ST6Gal-I and -II for *N*-/*O*-glycans, ST6GalNAc-I and -II for mucin *O*-glycans, and ST8Sia-II and -IV for polySia *N*-/*O*-glycans. Members of subgroup B are unique to sialylation in the glycolipids. They are ST3Gal-V for GM3, ST8Sia-I for GD3, and ST8Sia-V for GT3. Members of subgroup C are involved in the sialylation of both glycoproteins and glycolipids. They are ST3Gal-II, -III, and -IV; ST6GalNAc-III, -V, and -VI; ST8Sia-III and -VI (Figure 3).

All the STs utilize CMP-Sia as the donor substrate for adding Sia to the glycan chains. CMP-Sia is synthesized in the cytosol of mammalian cells. Sia molecules can be de novo synthesized from UDP-GlcNAc via *N*-acetylmannosamine (ManNAc) by a bi-functional enzyme, the UDP-*N*-acetylglucosamine 2-epimerase/*N*-acetylmannosamine-kinase (GNE), and the additional involvements of Neu5Ac-9-phosphate synthase (NANS) and Neu5Ac-9-Phosphate phosphatase [8]. The sources of the Sia can also be exogenous from the lysosomal degradation of the sialylated glycan chains and imported into the cytosol via the lysosomal transporter siallin (SLC17A5), or directly imported from extracellular space through ManT/SLC35Ax, the putative plasma membrane transporter of Sia. The cytosolic Sia is imported into the nucleus where it forms CMP-Sia by CMP-sialic acid synthetase (CMAS). After diffusing into the cytosol, CMP-Sia is transported into the Golgi lumen via SLC35A1 to become the substrate for STs.

Removals of the terminal Sia from the glycan chains are mediated by the sialidases (also called neuraminidases). They are widely found in vertebrates and microorganisms, but there are only four types of mammalian sialidases identified and characterized thus far: NEU1, NEU2, NEU3, and NEU4 [18,19,20,21,22,23,24,25]. NEU1 is predominantly localized in the lysosome, NEU2 is a soluble cytosolic enzyme, and NEU3 is in the endosomes and on the extracellular side of the plasma membrane. NEU4 has two isoforms, one in the mitochondria and the other in intracellular membranes. The intracellular localizations of these sialidases reflect the presence of the sialylated glycans in the glycoproteins or the gangliosides inside mammalian cells.

## 3. Charge–Charge Repulsion at Glycocalyx, Binding Receptors for Molecules, and Signaling as a Molecular Switch

The molecular complexity and extensive resource utilization in mammalian cells described above indicate the fundamental roles of sialylation in human biology and physiology. Moreover, from the perspective of evolution, sialic acids have an interesting expression distribution. They are found only in vertebrates, a few higher invertebrates, as well as gram-negative bacteria that are associated with human or animal hosts [6,120,121]. Disrupting the only sialyltransferase gene (*DsiaT*) in *Drosophila* results in pronounced locomotor defects, a progressive loss of coordination, and progressive temperature-sensitive paralysis [122], implying the most ancient functions of sialic acid in the nervous system [5]. In mammals, sialic acids are detected on most cell surfaces and secreted molecules, while the nervous system and immune cells have a very high abundance and diversity of sialoglycoconjugates [5,76]. They are the terminal sugars of many glycans within the glycocalyx on the cell surface that can extend outward 40–500 nm in mammalian cells [12]. The local concentration of sialic acids was found to be up to 110 mM in cells like lymphocytes [123]. 

**Figure 4:**
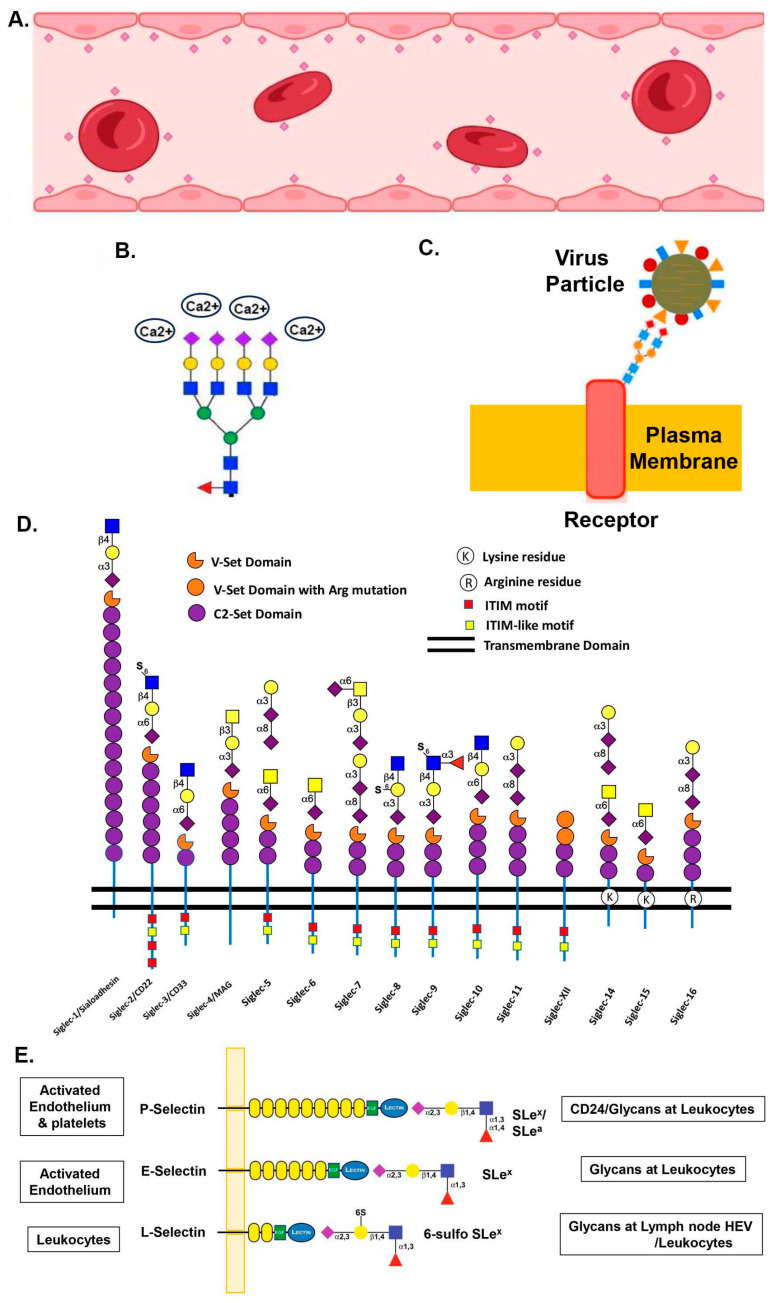
Functional modes for sialylation. (**A**) Negative charge repulsion “https://www.biorender.com (accessed on 4 November 2024)”. (**B**) Ca^2+^ binding [90]. Ca^2+^ ions (ovals) bind to the negatively-charged sialic acids (diamonds). (**C**) Receptors for viral and pathogen attachments [146]. (**D**) The human Siglec family receptors [91]. Functional domains, signaling motifs, and sialylated ligands are shown. Abbreviations: α3 = α-2,3 (for Neu5Ac), α-1,3 (for Gal and Fuc); α6 = α-2,6; α8 = α-2,8; β3 = β-1,3; β4 = β1,4; S = sulfated at C6. (**E**) Selectins mediate bindings to glycan capped with sialyl Lewis X (SLe^x^) structures [117]. Selectin domains and their expressed locations, sugar structures, and binding sites are shown.

The important roles of sialic acids in mammalian biology have been demonstrated in embryonic development. Schwarzkopf and colleagues have shown that inactivation of GNE by gene targeting causes early embryonic lethality in mice [124]. This is consistent with the fact that many cell adhesion molecules involved in early embryonic development are sialylated glycoproteins [125]. Interestingly, Abeln et al. also found that the complete loss of sialylation by CMAS deletion does not affect pluripotency and differentiation of mouse embryonic stem cells [126], suggesting that sialylation is more pivotal in the later stages of development. In human embryonic stem cells, sialylation is important in maintaining pluripotency and differentiation [127]. Not surprisingly, the expression of sialic acids changes during development and is altered in many human diseases [27]. All these combined features have made the concept of sialome (the total content of cellular sialic acids) a key controller for probing the structures and functions of the glycocalyx forests among various lateral domains and cell lineages [104]. As shown in Figure 4, five functional modes for sialylation can be summarized: (1) negative charge repulsion; (2) Ca^2+^ binding; (3) receptors for viral and pathogen attachments; and signaling as a molecular switch through the interaction with (4) Siglecs and (5) Selectins. 

The anionic and hydrophilic natures of sialic acids provide the most obvious functional role, as they can reduce nonspecific contacts between cells and components because of the charge repulsion (Figure 4A). Sialic acids on the luminal surfaces of vascular endothelia and blood cells prevent unwanted cellular interactions and impediment of circulation in the blood, whereas those on the foot processes of podocytes repel negatively charged plasma proteins into urinary ultrafiltrate in kidney glomerulus [27,90]. Charge repulsion from sialylation might affect the conformation of protein receptors like the epidermal growth factor receptor and consequentially modulate their activities [128,129]. Also, negatively charged sialic acids on the gangliosides can bind Ca^2+^ ions via electrostatic interactions at synapse at resting state but release the Ca^2+^ ions when an action potential arrives at the presynaptic terminals, modulating neurotransmission [90] (Figure 4B). Glycans terminated with sialic acids can also act as ligands for specific glycan-binding proteins (lectins) expressed in self-cells or pathogens [14,64,130]. As shown in Figure 4C, the Sia molecules on glycoproteins and gangliosides serve as entry receptors for a number of viruses, such as influenza viruses, adenoviruses, coronaviruses, rotaviruses, toroviruses, and reoviruses, as well as many bacterial toxins [reviewed by [97,130,131,132,133,134]] (Figure 4C). 

The most complicated yet fascinating function for sialic acids is their action as a molecular switch through the binding to various interacting partners. It is well known that sialylated glycans bind lectins such as Siglecs (Figure 4D) and Selectins (Figure 4E) for modulating cell signaling. Siglecs are receptors mainly expressed in the immune cells that recognize sialic acid-containing glycans with various binding preferences [62,75,82,91,98,100,101,135]. Currently, there are about 15 known human Siglecs, categorized into CD33-related Siglecs (Siglec-3/CD33, Siglec-5/CD170, Siglec-6/CD328, Siglec-7, Siglec-8, Siglec-9/CD329, Siglec-10/SLG2, Siglec-11, Siglec-XIII, Siglec-14, and Siglec-16) and the others (Siglec-1/CD169, Siglec-2/CD22, Siglec-4/MAG, and Siglec-15/CD33L3). The extracellular domains of these receptors typically contain the immunoglobulin (Ig) variable set domain (V) and the constant set domain (C2). While anchoring on a membrane through a transmembrane domain, most Siglecs consist of one or more cytosolic immune receptor tyrosine-based inhibitory motifs (ITIMs) to signal negatively to inhibit immune responses. Siglec-14, -15, and -16 do not have an intracellular domain but contain certain basic amino acids that recognize Tyr-based activation motif (ITAM)-encoding molecules (DaP-12) for signal activation [136]. By activating inhibitory SIGLEC receptors, the Sia molecules are pivotal checkpoint inhibitors. They function as a general “OFF” switch on many cellular processes, such as spinal axon outgrowth [137], microglial phagocytosis [76], myelin stability and neural sprouting after injury [76], osteoclast differentiation [138], osteoclast fusion [139,140], dendric cell function [141,142], and synovial fibroblasts pro-inflammatory transformation [143]. The Siglec proteins in immune cells also recognize Sia-containing glycans, and function in the innate and adaptive immune systems to allow discrimination between the “self” and “non-self” [reviewed by [64,144]]. Siglec-10 has recently become a crucial point of interest in modulating immune responses and facilitating immune escape in cancer, along with CD24 [145]. 

Selectins are cell-adhesion receptors that minimally recognize the sialylated glycans on *N*- and *O*-glycans of specific ligands [147,148] (Figure 4E). There are three types of Selectins, traditionally classified as P-, E-, and L-Selectins according to their expression on platelets, endothelial cells, and leukocytes, respectively. They are type-I membrane proteins consisting of an N-terminal lectin domain, an EGF-like module, and 2–9 consensus repeats, with distinct ligand specificities for sialyl Lewis^x^ (sLe^x^) for E-Selectin, sLe^a^ or sLe^x^ for P-Selectin, and sialyl 6-sulfo Le^x^ for L-Selectin. The interactions between Selectins and their ligands mediate the recruitment of leukocytes to sites of inflammation, homing of lymphocytes, and cancer metastases [77,117]. The combinatorial knockout of the STs involved in the synthesis of sLe^x^ decreased neutrophil rolling and lymphocyte homing [149]. The major functional roles of Selectins in cell adhesion of leukocytes, platelets, and hematopoietic progenitors cells along the vascular surface during injury or infection were extensively reviewed [147]. 

Sialic acids can also modulate signals through other molecular pathways. Through the binding of the regulatory complement factor H to sialic acids of the host cell glycocalyx, they can inhibit the complement responses by modulating key functions of the complement system during immune surveillance [76]. PolySia is another unique functional mechanism for Sia signaling. These macromolecules for Sia are highly expressed in the central nervous system and immune system, functioning for neural cell migration, axonal guidance, myelination, synapse formation, and plasticity of the nervous system, as well as immune cell migration and inflammation during innate immune response (reviewed by [5,9,45]). PolySia has an enormously hydrated volume and negative charge that can regulate the attraction and repulsion between cells [150,151]. PolySia on the cell surface forms a repulsive field that regulates cell–cell *trans* interactions negatively for increasing cell–cell interspace distance. They can also be an attractive field or reservoir for soluble molecules such as neurotransmitters and growth factors by condensing them on the cell surface [152]. PolySia on chemokine receptors can control immune cell trafficking by modulating the chemokine binding to the receptors [153].

The genetic knock-out (KO) phenotypes of STs provide a further mechanistic understanding of the sialylation pathways. Table 1 summarizes the published data on the genetic knockout of STs. Based on these phenotypes, four subgroups can be classified. Members of subgroup #1 are immune-related (*ST3Gal-I* KO, *ST3Gal-VI* KO, *ST6Gal-I* KO, *ST6GalNAc-III KO, ST6GalNAc-IV* KO, and *ST6GalNAc-IV* KO). The resulting genetic KO of these STs affected mature cytotoxic T lymphocytes [154,155] and impaired the leukocyte rolling and homing [149]. Decreased lymphocyte proliferation and IgM-producing levels [156,157], and reduced disialyl-T antigen levels [158]. Members of subgroup #2 are metabolic-related (*ST3Gal-II* KO, *ST3Gal-IV* KO, *ST3Gal-V* KO, and *ST6GalNAc-V* KO). The KOs can have a beneficial phenotype with late-onset obesity and insulin resistance [159,160], resulting in thrombocytopenia and bleeding disorder [149,161], and increased sensitivity to insulin but also with a loss of hearing [162,163] and enhanced tumor growth and angiogenesis [164], as well as corresponding human mutations with coronary artery disease [165]. Members of subgroup #3 are neurological-related (*ST3Gal-III* KO, *ST8Sia-I* KO, *ST8Sia-II* KO, and *ST8Sia-III* KO). The KOs can prevent >95% GSL sialylation [166], display a sudden death phenotype, and are susceptible to the induction of lethal seizures by sound stimulus [167], defects in the formation of neurobiological synapses [168], desialylating several striatum-enriched G-protein-coupled receptors (GPCRs) possibly related to several basal ganglia diseases [such as schizophrenia and Parkinson’s disease (PD)] [169]. Members of subgroup #4 are miscellaneous (*ST6Gal-II* KO and *ST6GalNAc-I* KO). *ST6Gal-II* KO showed no efficient enzyme activity in vivo [170]. *ST6GalNAc-I* KO reduced sialylated glycans on the ocular mucins [171]. Reports on the *ST6GalNAc-II* KO, *ST8Sia-V* KO, and *ST8Sia-VI* KO are currently not available. Although none of the *ST* knockouts described above are lethal, the diversity of the phenotypes reflects the complexity of STs’ biological functions. 

## 4. Sialic Acid Related Human Diseases

Given the essential roles of sialic acids in mammalian biology, it is not surprising that aberrant sialylation can lead to serious diseases (summarized in Table 2). These disorders range from defecting genes involved in sialic acid metabolism to anabolic and catabolic deficits, autoimmunity against sialylated self-molecules, dysregulation of polySia in association with psychiatric disorders and neurodegeneration, malfunction of sialic acid binding proteins Selectin in cardiovascular disease, and wide-spread aberrant sialylation associated with various types of cancer and malignant transformation.

### 4.1. Infectious Disease

Sialic acids are the known targets for many pathogenic organisms and toxins [20,27,97,130,134,174]. Influenza viruses employ the surface homotetrameric sialidase to release new viral particles from infected cells by cleaving the host surface sialic acids [20,130]. Three anti-influenza drugs, oseltamiviur [Tamiflu^TM^, Roche (Basel, Switzerland) and Gilead (Foster City, CA, USA)], zanamivir (Relenza^TM^, GlaxoSmithKline, Brentford, UK), and peramivir (Rapivab^TM^, BioCryst, Durham, NC, USA) received FDA approval (Table 3). 

Several Paramyxodivirdae family viruses, such as the Newcastle disease virus, Parainfluenza viruses, Mumps virus, and Sendai virus, have viral genes that encode a haemagglutinin-sialidase protein [20]. Infections with the influenza virus are through the binding of trimeric viral haemagglutinin (HA) proteins to α2,6-linked sialic acid on human glycans [175]. Reovirus infection is common in humans but with disease restricted to the very young population. The attachment of reovirus to host cells is via the binding of the trimeric outer-capsid protein σ1 to ganglioside GM2 (T1 serotype) [176] or α2,3- α2,6- α2,8 linked sialylated glycans (T3 serotype) [177]. Adenoviruses infect humans, causing symptoms of conjunctivitis or upper respiratory illness with some strains or in immune-compromised individuals. The virus binding to human erythrocytes is mediated by the trimeric species D adenovirus 37 (Ad37) to the ganglioside GD1 [178]. Rotaviruses, the leading cause of childhood diarrhea worldwide, attach human cells through the trimeric outer-capsid protein VP4 to the ganglioside GM3 [179]. Human coronavirus-OC43 and -HKU1 are known to employ 9-O-aceylated-Sia as the entry receptors [180]. Recently, it has been reported that monosialylated gangliosides such as GM1, GM2, and GM3 mediated binding and viral entry of severe acute respiratory syndrome coronavirus-2 (SARS-CoV-2) [181]. Consistently, multivalent sialic acid glycoclusters were found to be potent inhibitors for SARS-CoV-2 infection [182]. NEU1 was shown to regulate SARS-CoV-2 replication and a newly developed NEU1 inhibitor dramatically reduced viral replication in vitro [183]. The human cell surface sialome has been affected by SARS-CoV-2-related pathology [96]. In addition, human Bufavirus-1 from patients exhibiting acute diarrhea also binds to terminal sialic acids through its capsid surface [184], suggesting a possible anti-pathogenic parvoviruses strategy. Sialidase inhibitors were found to suppress mumps virus replication and infection [185].

**Table 2:** Human diseases related to sialic acids.

Disease Types	Sialic Acid Pathways	References
Sialuria, an autosomal dominant disorder Neuromuscular disorders, recessively inherited disease hereditary inclusions body myopathy (HIBM)	GNE mutations not inhibited by CMP-sialic acid (R263L, R266Q, R266W), increased excretion of free sialic acids; NANS.Mutations in GNE over both epimerase and kinase moieties	[186,187,188,189]
Lysosomal storage diseases	ST3Gal-V, B4GalNT1, GNE, SLC17A5	[51,54]
Cancer and malignant transformation	Overexpression of various STs, Siglecs, Selectins, GM3, GD2, GD3,	[13,14,30,31,33,36,38,39,40,47,49,55,69,70,71,72,73,74,75]
Autoimmunity and Inflammation diseases	ST3Gal-V, Siglecs, Sialidases, Selectins, GM1, GM3, GD1,GD1a, GD1b, GD3, GT1a, GT1b, GQ1b	[14,28,36,61,62]
Cardiovascular and Metabolic diseases	ST3Gal5 for GM3, neuraminidase1–4, and inhibiting sialyltransferases for atherosclerosis	[87,88]
psychiatric disorders and neurodegeneration	GM1, GM3, GD1b, GD3, GT1b, ST8-Sia-II, Siglecs	[9,48,50,51,53,89,90,91,92,93]
Infectious diseases	Viral or human Sialidases and STs, GM1, GM2, GD1	[20,130]

**Table 3:** Summary of sialic acid-related approved products.

Targets	Product Names	Disease Indications	Marketing Approval
Influenza’s neuraminidase	oseltamivir (Tamiflu^®^)	Treat and prevent Flu (Influenza A and B viruses)	US FDA 1999, Roche and Gilead
Influenza’s neuraminidase	zanamivir (Relenza^®^)	Treat and prevent Flu (Influenza A and B viruses)	US FDA 1999, GlaxoSmithKline
Influenza’s neuraminidase	peramivir (Rapivab^®^)	Treat and prevent Flu (Influenza A and B viruses)	US FDA emergency use authorization in 2009, and full approval in 2014, BioCryst
CD33 (Siglec-3)	gemtuzumab ozogamicin (Mylotarg^®^)	Acute myeloid leukemia	US FDA first approved in 2000, withdrawn in 2010, and re-approved in 2017, Pfizer (New York, NY, USA)
CD22 (Siglec-2)	inotuzumab ozogamicin (Besponsa^®^)	Relapsed or refractory B-cell precursor acute lymphoblastic leukemia	US FDA 2017, Pfizer
CD22 (Siglec-2)	moxetumomab pasudotox (Lumoxiti^TM^)	relapsed or refractory hairy cell leukemia	US FDA 2018, withdrawn in 2022, Astrazeneca (Cambridge, UK)
P-Selectin	Crizanlizumab (Adakveo^®^)	Sickle cell disease	US FDA 2019, Novartis (Basel, Switzerland)
Glycolipid GD2	Dinutuximab (Unituxin^®^)	second-line treatment for children with high-risk neuroblastoma	US FDA 2015, EMA withdrew 2017 United Therapeutics (Silver Spring, MD, USA)
Glycolipid GD2	dinutuximab beta (Qarziba^®^)	second-line treatment for children with high-risk neuroblastoma	EMA 2017, United Therapeutics
Glycolipid GD2	Naxitamab (Danyelza^®^)	Treatment of neuroblastoma, osteosarcoma, and other GD2-positive cancers	US FDA 2020, Y-mabs Therapeutics (New York, NY, USA)
Uridine diphosphate-glucose ceramide glycosyltransferase	Eliglustat (Cerdelga^®^)	Gaucher’s disease treatment	US FDA in 2014, Sanofi-Genzyme (Cambridge, MA, USA)
Anti-idiotypic mouse monoclonal antibody that mimics N-glycolyl-gangliosides	Racotumomab (Vaxira^®^)	Antibody therapeutic vaccine induces an immune response against NeugcGM3 present in tumor cells for the treatment of recurrent or advanced NSCLC	Approved in Argentina (Elea Laboratories) and Cuba (CIM)

Trypanosome which can cause extremely debilitating and life-threatening illnesses, i.e., African sleeping sickness and Chagas disease, has genes that encode the trans-sialidase that can cleave sialic acids from host cells and then covalently attach them to terminal β-galactose acceptor molecules on the surface of the parasite. This modification can effectively mask itself in pseudo-host tissue [190]. Multiple pathogenic bacterial species, such as *Vibrio cholera*, *Corynebacterium diptheriae*, and *Streptococcus pneumoniae*, can also utilize sialic acids for pathogenicity [191]. In addition, a handful of pathogenic bacteria can express STs as virulence factors to produce molecular mimics of mammalian glycans, which could evade the host immune system by adding terminal sialic acid residues to their lipopolysaccharides [192]. Siglec molecules like Siglec-7 can also serve as a major entry receptor for viruses, through which varicella-zoster virus infects human monocytes by interacting with viral glycoprotein B [193]. Hepatitis B viruses (HBV) bind to human Siglec-3 through α2,6 sialoglycans of the HBV surface antigen [194]. All these observed mechanisms could potentially be targets for therapeutic intervention.

### 4.2. Autoimmunity and Inflammation

In autoimmunity, hyposialylation is key for chronic inflammation, the anarchic activation of the immune system, and organ lesions [28]. This is consistent with the general “OFF” functional signal imposed by sialic acids. Sialylation modulates the maturation and activation of dendritic cells (DC) [195] and Siglec-2 (CD22) is an important negative regulator for B-cell activity [145]. The sialylated motifs on the leukocyte surface modulate the trafficking to secondary lymphoid organs and the inflammatory sites through the binding to selectins [196]. It has been reported that polySia on CCR7 (C-C chemokine receptor type 7) for chemokine controls DC trafficking by regulating chemokine recognition [153]. In addition, Th1 and Th17 cells lacking sialyation are vulnerable to galectin-1-mediated cell death, whereas mice deficient in galectin-1 developed autoimmune inflammation [197]. The hyposialylation of anti-proteinase-3 IgG has been shown to correlate with the severity of granulomatosis with polyangiitis, a small-sized vessel necrotizing vasculitis [198]. Therefore, increasing sialylation in IgG or autoantibodies has been proposed as a promising strategy to treat autoimmune diseases [28]. Moreover, desialylation of IgE attenuates effector-cell degranulation and anaphylaxis in several functional models of allergic disease [199,200]. T-cell-specific deletion in mice indicates that sialic acids on the T cells are important for their survival and maintenance [201]. Sialic acids in B cells were shown to be crucial for survival and apoptosis protection [202].

The production of anti-sialic acid antibodies can lead to the development of autoimmune diseases. Anti-Neu5Gc antibodies were found in patients with autoimmune Hashimoto’s thyroiditis [203], as Neu5Gc is not normally produced by humans [204]. Likewise, Neu5Gc is found to trigger autoimmunity termed “xenosialitis” [205]. The autoantibodies can be produced spontaneously from the natural autoantibody repertoire such as IgM antibodies or induced by infections that share structurally similar glycans to the gangliosides. The surface lipo-oligosaccharides of *Campylobacter jejuni* display molecular mimicry to GM1, GD1a, GT1a, and other gangliosides. The autoantibodies can activate complement and recruit macrophages, causing structural and functional disorganization of nerve conduction. A large subset of cases of Guillain–Barre syndromes (GBSs), the leading cause of acute neuromuscular paralysis, involve anti-sialylated glycolipids [206]. Both subsets of AMAN (acute motor axonal neuropathy) and MFS (Miller-Fisher Syndrome) are caused by the immune crossover between ganglioside-like epitopes on pathogens and endogenous gangliosides on axons and nerve terminals. The surface of lipooligosaccharides of *C. jejuni* strains associated with GBS mimicked the structures of human gangliosides such as GM1 and GD1 in AMAN, and GQ1b in MFS [5]. GM3S (*ST3Gal-V*)-KO mice exhibited exacerbated inflammatory arthritis in the rheumatoid arthritis (RA) mouse model [207], suggesting an essential role of GM3 in the pathogenesis and progression of RA. In addition, sialylation on platelet surface receptors correlated with the clearance of platelets which was shown to be a main cause for thrombocytopenia being a complication in immune disorders [208,209]. Administration of GM1, GD3, GD1a, GD1b, and GT1b could decrease inflammatory microglia responses in vitro, while GM3 and GQ1b displayed proinflammatory activity [210], indicating that the gangliosides are important modulators of microglia inflammatory responses. GM1, GM3, and GD3 also have a direct implication in podocytopathies such as IgA nephropathy, membranous nephropathy, and lupus nephritis [60].

### 4.3. Psychiatric Disorders and Neurodegeneration

It is well known that sialic acids are expressed at the highest levels in the brain where they regulate neuronal sprouting, axon myelination, neuronal connection remodeling, as well as microglial homeostasis. Significant progress has been made in the understanding of the neural function of sialic acids and their relationships with brain health and neurodegeneration diseases [9,48,50,51,53,76,89,90,91,92,93]. The sialic acid–Siglec interaction systems prevent the excessive and detrimental immune responses, yet the malfunction of interaction with Siglec3/CD33, Siglec-11, and Siglec-14 have been associated with neurodegenerative diseases such as Alzheimer’s disease (AD) [53,89,90,91]. In addition, psychiatric disorders, such as schizophrenia, bipolar disorders, and autism spectrum disorders, have been found to be linked to the polysialyltransferase ST8Sia-II and Siglec-4/MAG [9,50,92]. Furthermore, neurodegenerative diseases and mental health disorders such as PD and Huntington’s disease (HD) are closely associated with altered ganglioside expression [48,92].

The elevated levels of serum sialic acids found in AD patients [211] suggest various roles for sialic acids in AD pathologies. The extracellular amyloid beta (Aβ)1–40 can bind to different gangliosides and change its secondary structure, likely through the involvements of electrostatic forces between negatively charged sialic acid and the Aβ [90,212], suggesting disrupting the ganglioside-Aβ interaction could decrease the Aβ deposition in AD brains. Consistent with these findings, CD33 was recently identified as a genetic risk factor for late-onset AD [213]. Evidence supports that Aβ plaque can avoid microglia-mediated clearance with the aid of the sialic acid–CD33 interaction [214].

Dysregulation of polySia and NCAM has been associated with psychiatric disorders, such as schizophrenia, bipolar disorder, and autism [5,9]. The relationship between polySia and neurodegeneration, namely PD, AD, and HD, has also been established [5,9]. Genome-wide association studies (GWASs) identified variants of ST8SIA2 or the loss-of-function mutations associated with psychiatric conditions such as schizophrenia, bipolar disorder, and autism [215,216,217]. Consistently, polySia expression was reduced in patients with schizophrenia [218]. In multiple sclerosis, polySia seems to have an inhibitory role in myelination as it is preferentially expressed in demyelinated axons, and not in remyelinated axons [219]. Intravitreal injection of polySia can limit tissue damage in a model of age-related macular degeneration by restraining phagocyte activity and complement activation [220], implying a potential therapeutic application for curbing nervous system damage. Polysialyltransferase (ST8Sia-II) was reported to be a potential susceptibility gene for several mental illnesses such as bipolar disorder and schizophrenia [215].

Systemic deficiency in GM1 in the brain and all peripheral tissues has been found in PD and HD, whereas the gangliosides with b-series (GD1b, GT1b) are associated with AD [48,50]. Complex gangliosides such as GM1 are significantly less in the brains of AD patients than healthy subjects [221]. Patients who carry rare mutations in the *ST3Gal-V* gene develop the infantile-onset symptomatic epilepsy syndrome and exhibit refractory epilepsy accompanied by neurological deterioration [59,222]. Loss-of-function in B4GALNT1 that blocks the downstream biosynthesis of GM3 and GD3 can result in a complicated form of hereditary spastic paraplegia [223]. Inactivation mutations in ST3Gal-III that are involved in the synthesis of GD1a and GT1b were found in patients with West syndrome (an infantile epilepsy disorder) [224]. *GD3S(ST8Sia-I)-KO* that increased GM1 could improve memory and reduce amyloid-beta plaque for preventing AD [225]. In addition, GM1 and other sphingolipids can be bound by α-synuclein, which inhibits fibril formation, the neuropathologic characteristic of PD degenerating dopaminergic neurons [51]. Supplementations of GM1 or GD3 via intranasal infusion [226] could trigger the expression of neuroprotection genes and exert neuroprotective effects [227]. Infusion of GQ1b was also found beneficial in an AD mouse model [228]. In HD, a reduction in GM1 in the patients was also observed [229]. GM1 restoration showed therapeutic benefits in a mouse model [230], implying a GM1-mediated reduction in the huntingtin aggregates as a potential treatment approach. Administration of GM1 is protective against glutamate-excitotoxicity in the disease-affecting motor neurons for amyotrophic lateral sclerosis (ALS) [231,232] and stroke [59]. Infusion of exogenous GM3 can also slow down disease progression and prolong ALS mouse survival [231]. Administration of GM4 and GD1a can induce the remyelination process in the multiple sclerosis model [233]. 

### 4.4. Cardiovascular and Metabolic Diseases

The interaction of Selectins expressed on activated endothelium with SLe^x^-capped *N*- and *O*-glycans of Selectin ligands on leukocytes is deregulated in diseases like atherosclerosis and sepsis [117]. Atherosclerotic lesion formation is facilitated by the participation of Selectins, and Selectin knockout prevented acute inflammation and reduced atherosclerotic lesions in mice [234,235]. The roles of sialic acids in the low-density lipoproteins are implied in atherosclerosis development because of the reported role in endothelium lipid uptake determination [27,236,237]. Several alleles of P- and E-Selectin are found to be associated with cardiovascular risk [238,239].

The sialidase genes NEU1 and NEU3 were found upregulated in the cardiovascular disease models [240], whereas disrupting *NEU1* and *NEU3* in mice attenuates atherosclerosis, cardiac hypertrophy, and cardiac fibrosis [241,242]. NEU1 was found upregulated in idiopathic pulmonary fibrosis (IPF) [243]. Eight of nine IPF patients had a high sialidase activity in the lung bronchoalveolar lavage fluid, while no such activity was detected in the healthy control [244]. Sialidase inhibitors have therefore been proposed as potential therapeutics for fibrosis, as NEU3 is found necessary and sufficient for pulmonary fibrosis through the transforming growth factor-β1 pathway [88]. In addition, NEU1 can potentiate inflammation in atherosclerosis [245]. The Sialidases were found to play important roles in the inflammatory response after cardiac stress and are also potential therapeutic targets for cardiac disease [25]. In addition, desialylated LDL is shown to be highly immunogenic and can induce the production of proatherogenic autoantibodies [246,247]. Consistently, the hyposialylation of lipoproteins, modulating by Sialidases, was found closely associated with cardiovascular diseases such as atherosclerosis [87]. Moreover, desialylation of voltage-gated ion channels can lead to impaired voltage-gated Na^+^ channel function through genetic knockout [248]. Decreased sialylation in isoform-specific voltage-gated K^+^ channels also correlated with the adverse effects on cardiomyocyte electrical signaling [249]. In *ST3Gal-IV* knockout mice, their hearts developed dilated cardiomyopathy [250]. The observations above suggest that increasing sialyation might provide potential therapeutic benefits for cardiovascular diseases.

Sialylation in the gangliosides has also been shown to play a potential role in metabolic diseases. GM3S (*ST3Gal-V*)-KO mice display higher insulin sensitivity [162,251], protecting them from high-fat-diet-induced insulin resistance. GM3S gene-deficiency ameliorates the obese phenotype when given high-fat diets [252]. GM3 ganglioside might be a negative regulator of insulin signaling and a therapeutic target in type 2 diabetes [48]. Therefore, targeting the GM3 biosynthesis pathway represents a potential therapeutic strategy. In addition, it was shown that polySia nanoparticles can attenuate the complement factor H alternative pathway, which is implicated in the etiology of age-related macular degeneration [253].

### 4.5. Cancer and Malignant Transformation

Sialic acid expression is widely upregulated in various types and stage cancers, and is the distinctive feature associated with malignant properties including invasiveness and metastatic potential [27,33,70,71,77,254]. Aberrant sialylation plays a vital role in cancer cell growth and survival, promoting cancer chemoresistance, tumor invasion and migration, and enhanced immune evasion [70,77]. Hypersialylation in cancers can be attributed to both the upregulation of STs as well as the downregulation of the Sialidases. PolySia expression has also been found strongly correlated with several highly metastatic cancers such as lung carcinoma, neuroblastoma, and pancreatic cancer [255]. E-selectin has been shown to facilitate the entry of breast cancer cells into bone marrow [256], promoting cancer metastasis. Hypersialylation of cell death receptors such as Fas and tumor necrosis factor receptor 1 can enable cancer cells to resist cell death [257]. Hypersialylation can also enable cancer cells to avoid detection and destruction by the immune system, which is mainly mediated by Siglecs [258]. Sialylation is a cloak for tumors to trick the immune system in the microenvironment [72]. Increased sialylation of cancer cells has been linked to the treatment resistance of chemotherapy [259,260]. Prostate cancer cells resistant to the hormone therapy enzalutamide were found to increase expression of ST6Gal-I, and their resistance can be reversed by treatment with sialyltransferase inhibitor P-SiaFNEtoc [261]. ST6Gal-I and α2,6Sia were also shown to promote prostate tumor growth and invasion [262]. Cultured pancreatic cancer cells exhibited significantly higher levels of α2,6Sia modification with an enhanced reproductive capacity, and are less influenced by the immune cell landscape within the tumor microenvironment [263]. 

Transcriptomic data reveal that high SIGLEC-7 expression predicts a poor prognosis in glioma patients and is closely associated with M2 macrophages in the tumor environment [264]. Siglec-15 has been proposed as a new class of immune inhibitors with potential implications in anti-PD-1/PD-L1 resistant patients [265]. Antibodies blocking Siglec-15 can inhibit the spread of metastatic cancer cells from bone lesions to other organs [266]. Anti-Siglec-7 or NK cell engagers through Siglec-7 demonstrated robust in vitro killing of ovarian cancer and improved survival in challenged mice [267]. Siglec-9 has been shown to act as an immune-checkpoint molecule on macrophages in glioblastoma that can be targeted to enhance the anti-PD-1/PD-L1 therapeutic efficacy [268]. Pancreatic ductal adenocarcinoma tumor cells drove tumor-associated macrophage differentiation via Siglec-7 and Siglec-9 [269]. Blocking Siglec-9 could also improve the antibody-mediated neutrophil cytotoxicity towards tumor cells [270]. Siglec-5 was found to be an inhibitory immune checkpoint molecule for the tumor-specific activation of human T cells [271]. The interaction between sialic acids and Siglec-5/14 hindered the CD11b/CD18-regulated binding between neutrophils and antibody-opsonized tumor cells, representing a target for innate checkpoint blockage in the tumor microenvironment [272]. Siglec-6 was shown to be a new target for CAR T-cell therapy in acute myeloid leukemia [273]. Siglec-10 was recently identified as a new dendritic cell checkpoint for cervical cancer immunotherapy [274].

Circulating gangliosides shed from tumor cells have long been known to correlate with tumor progression and relapse [275], i.e., GM3 in the serum of breast cancer patients [276], and GD2 for neuroblastoma [277]. It has also been proposed that gangliosides could affect angiogenesis for tumor development, as GD3 concentration is more than 50% in anaplastic gangliogliomas versus <10% in healthy brain tissue [278]. It has been shown that GD2 is highly expressed in cancer stem cells, therefore the upregulation of GD2 promotes tumor initiation and progression [47]. GD3 synthase (ST8Sia-I), regulating the biosynthesis of GD3 and GD2, is found upregulated in most tumors including breast cancer, melanoma, neuroblastoma, and hematological malignancy [47]. The roles of sialylation in cancers have been reviewed by a number of recent articles [13,14,30,31,33,36,38,39,40,41,47,49,55,69,70,71,72,73,74,75,76,77].

### 4.6. Free Sialic Acid Storage Diseases and Lysosomal Storage Diseases

Sialylation is directly involved in a number of genetic diseases, including free sialic acid storage disease and lysosomal storage diseases (LSD). Lysosomal free sialic acid storage disorder is an extremely rare autosomal recessive multisystem disorder caused by defects in the lysosomal sialic acid membrane exporter SLC17A5 [34]. Its defects cause enlarged lysosomes and 10–100-fold increased urinary excretion of free sialic acids with clinical symptoms of coarse facial features and progressive neurodegenerative features. Furthermore, Sialuria is another type of sialic acid storage disease [186,187,188] in which GNE, the essential gene for sialic acid synthesis, is no longer inhibited by its end products. On the other hand, bi-allelic mutations in NANS were found in people with infantile-onset severe development delay and skeletal dysplasia [279], supporting the essential roles of sialic acids. 

Congenital disorders of glycosylation (CDG) type I are caused by defects in the formation of the glycan structure on the glycolipid dolichol-glycan precursor prior to the attachment to the Asn residue, whereas type II involve abnormalities in the control of the N-linked branching structures on the glycoproteins [280,281]. CGDs typically affect many muscular, developmental, and neurological functions [36]. In LSDs such as Niemann–Pick disease types A, B, and C, the levels of GM2 and GlcCer increase due to accumulating sphingomyelin and cholesterol [282]. Due to the inhibition of gangliosides catabolism by mucopolysaccharides in LSDs, gangliosides GM1, GM2, and GM3 accumulate in Hurler, Hunter, Sanfilippo, and Sly syndromes [282]. 

Disrupting the GlcCer biosynthetic pathway has been found to be lethal in mice development, but the loss of certain complex ganglioside structures is tolerable, resulting in neurological dysfunction [283]. The *ST3Gal-V* mutation for GM3-synthase, converting LacCer to GM3, causes a congenital infantile-onset seizure disorder accompanied by profound developmental stagnation [222,284]. *B4GalNT1* (GM2 synthase) deficiency interrupts the synthesis of a-series gangliosides and can cause spastic paraplegia, which is progressive lower limb weakness and spasticity [285,286]. More than 70 LSDs that are related to the ganglioside lipid have been reported [283,287]. In addition, genetic deficiency of Sialidase *NEU1* can result in sialidosis types 1 and 2 [288].

## 5. Therapeutic Applications of Sialic Acid and Clinical Developments of Sialic Acid-Related Therapies

Besides the best-known sialic-acid-related drugs for inhibiting influenza neuraminidase, active therapeutic research and development on sialylation includes targeting Selectins for immunology, Siglecs for cancer, as well as vaccines and antibodies targeting mammalian sialic acids. Table 3 summarizes the current twelve approved products on the market, with a significant number of candidates in clinical development. Important examples are highlighted below.

Rivipansel (GM1-1070), GlycoMimetics’ pan-Selectin inhibitor, is capable of binding to E-selectin, P-selectin, and L-selectin with micromolar affinity, therefore inhibiting leukocyte rolling and reversing vascular occlusion in a mouse model of sickle cell disease [289]. Rivipansel, containing moieties analogous to the sLe^x^ tetrasaccharide and the carboxyl acid of sialic acid, could reduce the time to resolution of vaso-occlusion and decrease opioid use for patients with acute sickling crises in a phase II trial (NCT01119833) [290]. Although the phase III trial (NCT02187003) of rivipansel in sickle cell disease did not meet its primary end points, the post hoc analysis reveals that the timing of rivipansel administration after pain onset might be critical to achieving efficacy and benefit [291]. As summarized recently [14], other similar small-molecule inhibitors for Selectins are either in the early stages of clinical development or terminated.

For biological Selectin inhibitors, a chimeric P-selectin-immunoglobulin fusion protein, rPSGL-Ig, was developed to promote thrombolysis and to prevent reocclusion in porcine models of thrombolysis and myocardial infarction [292]. A phase II study (NCT00876902) of liver allograft function showed improved liver function by rPSGL-Ig after transplant [293], yet a phase IIa study (NCT00298168) for renal allograft showed no efficacy [294]. Uproleselan (GMI-1271) is an E-selectin-specific inhibitor that showed promising benefits in combination with chemotherapy for AML in a phase I/II trial [295], and a clinically meaningful improvement in median overall survival in a phase III trial for relapsed/refractory “https://glycomimetics.com (accessed on 4 November 2024)”, leading to the FDA breakthrough and fast-track designations.

Selectin-antagonizing antibodies have met much better success. A P-selectin blocking antibody crizanlizumab was developed as a prophylactic agent for vaso-occlusive crises in patients with sickle cell disease to reduce the frequency of pain crises in a phase II trial (NCT01895361) [296]. The trial results [297,298] won crizanlizumab FDA approval in 2019. Crizanlizumab expanded into retinal vasculopathy with cerebral leukoencephalopathy and showed preliminary efficacy in a phase II trial (NCT04611880) [299]. Another P-selectin antagonist antibody (inclaclumab) was developed and shown to reduce myocardial damage in patients undergoing percutaneous coronary intervention for non-ST segment elevation myocardial infraction in a phase II study (NCT01327183) [300]. However, another phase II trial (NCT01245634) in patients undergoing coronary artery bypass graft surgery did not show efficacy [301].

The beginning of therapies targeting Siglecs can be traced back to the development of the FDA-approved antibody-drug-conjugates gemtuzumab ozogamicin and inotuzumab ozogamicin target CD33 and CD22, respectively, for blood tumors. Recently, a Siglec-15 blocking antibody (NC318) showed clinical activity in patients with advanced PD-1 Axis inhibitor refractory NSCLC in a phase II trial (NCT03665285) “www.nextcure.com (accessed on 4 November 2024)”. Soluble CD24 with Fc domain (CD24Fc) is a Siglec-10 agonist [302], which is now in a phase III trial for the prevention of acute graft-versus-host disease (NCT04095858) and inflammation associated with severe COVID-19 (NCT04317040), as well as a phase I/II trial (NCT04060407) for immune-related adverse events associated with checkpoint inhibitor therapy [14]. An anti-Siglec-8 antibody lirentelimab (AK002) developed by Allakos was shown to be effective in treating eosinophilic gastritis and duodenitis in a phase II study (NCT03496571) [303,304]. Alector developed an anti-CD33 antibody, which is currently in a phase I clinical trial (NCT03822208) as a novel therapeutic strategy for AD. Intravitreal application of oligo- and polysialic acid linked to a poly(lactic-co-glycolic acid)(PLGA)-poly(ethylene glycol)(PEG) backbone is currently in phase II trials as a treatment for geographic atrophy [92], although the inflammatory side effects of PLGA/PEG might interfere with long-term eye applications.

A Gangliosides GM2-KLH (keyhole limpet hemocyanin) conjugate was developed as a tumor vaccine candidate [305], yet a phase III trial (NCT00005052) in patients with stage II melanoma did not show benefits [306]. A sialyl-Tn (sTn)-KLH conjugate vaccine (Theratope) [307,308] was developed to promote the generation of anti-sTn antibodies in breast cancer patients. However, a phase III trial (NCT00003638) in metastatic cancer patients showed no efficacy [309]. A heptavalent vaccine including GM2 and sTn linked to a single KLH scaffold was in a phase I trial (NCT01248273) with a demonstrated safety [310]. A bivalent vaccine composed of GD2 and GD3 conjugated to the KLH is in a phase II trial (NCT00911560) for high-risk neuroblastoma [311].

The anti-GD2 antibody dinutuximab was approved for the treatment of high-risk pediatric neuroblastoma [312], based on the result of a landmark phase III (NCT02641782) with improved overall survival [313]. An anti-GD2 antibody, hu14.18, fused with interleukin-2, was tested in phase II studies in patients with R/R neuroblastoma [314,315]. An anti-GD2 trispecific T-cell engager antibody (OKT3 x hu3F8) was tested in a phase I/II study in patients with GD2-positive tumors [55]. A fully human anti-Fc-GM1 antibody was developed for use in small-cell lung cancer [316], and tested in a phase I/II trial for small-cell lung cancer [317] (NCT02247349). A CAR-T cell against GD2 has been advanced to human trials in neuroblastoma [318] and glioma [319]. Since gangliosides are thought to affect angiogenesis of tumor development, inhibiting the GSL synthesis might also represent an effective immunotherapy approach, i.e., the combination of eliglustat and an anti-PD-1 antibody for the treatment of advanced cancers (NCT04944888) [320].

An anti-GD2 CART-NKT cell therapy is in a phase I trial (NCT03294954) for R/R neuroblastoma [321], one of three patients showing an objective response with regression of bone metastatic lesions. Another GD2 CAR-T cell therapy is in a phase I trial (NCT04196413) for diffuse intrinsic pontine glioma or diffuse midline gliomas [322], with three out of four patients obtaining clinical benefits without signs or symptoms of on-target and off-tumor toxicity. A GD2/GD3-based vaccine was tested in a small phase I trial for high-risk neuroblastoma and recently proceeded to a phase II study (NCT00911560) [323]. The pharmacological use of exogenous GM1 has long been investigated in clinical studies with demonstrated safety [324], yet its significant therapeutic benefits need to be further studied for neurodegenerative diseases [59].

An anti-HER2 antibody-sialidase conjugate that can de-sialylate cancer cells and prolong survival times in mice [325] is currently in phase I/II clinical trials (NCT05259696) “https://palleonpharma.com (accessed on 4 November 2024)”. DAS181, a sialidase fusion is currently in a Phase III clinical trial for the treatment of lower respiratory tract parainfluenza infection in immunocompromised patients [NCT03808922, “www.clinicaltrials.gov (accessed on 4 November 2024)”] [97], leading to FDA fast-track and breakthrough designations. Preclinical models showed fusing a sialidase to a bispecific T-cell engager enhanced the potency of therapies in leukemia, melanoma, and breast cancers [326]. Several monoclonal antibodies against the HA protein stalk region have been studied in clinical studies of phase II or III in hospitalized influenza-A-infected patients [97]. 

AMG330, a human bispecific T-cell engager (BiTE) against CD33/CD3, produced a potent T-cell cytotoxic response against primary AML [327] and was tested in a phase I trial (NCT02520427). AMG673, an anti-CD33/CD3 half-life extended BiTE, was tested in a phase I trial (NCT03144245) [75]. An anti-CD33/CD3 bispecific antibody (JNJ-67571244) was developed to recognize the C2 domain of CD33 to overcome the polymorphism of CD33 and is currently in a phase I trial [328]. An actinium-225 conjugated anti-CD33 antibody lintuzumab has been tested in patients with acute myeloid leukemia with safety and some extent of efficacy in a phase I trial [329]. An anti-CD22 CART cell was tested in a phase I trial for R/R Pre-B cell acute lymphoblastic leukemia (NCT02315612) [330], establishing clinical activities. Other anti-CD22 clinical-staged therapeutic candidates include Epratuzumab for diffuse large B-cell lymphoma and indolent non-Hodgkin lymphoma [331,332], and moxetumomab pasudotox (Lumoxiti^TM^) which showed efficacy in hairy cell leukemia [333]. 

## 6. Future Perspectives

**Figure 5:**
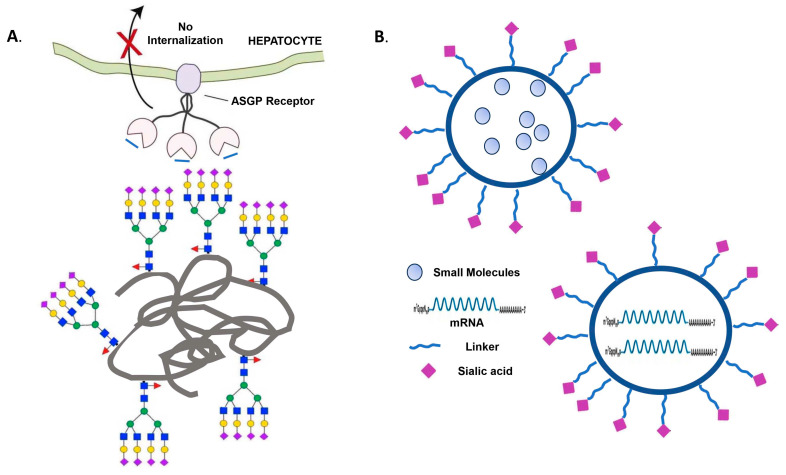
Therapeutic applications with sialylation. (**A**) Long-acting biotherapeutics with engineered sialylation [338]. Sialylated glycoproteins are not recognized by asialoglycoprotein (ASGP) receptors in the liver and are thus protected from uptake and degradation. (**B**) Sialic acid-modified micelles or lipid nucleotide particles that bind to E-selectin in tumor tissues [106]. The drug-loaded micelles or mRNA-containing LNPs can be coated with sialic acids for site targeting.

The important and exciting roles of sialic acids in biological functions have been established and extensively investigated over the past three decades. Many new technologies related to sialic acids are in development. For instance, sialic acid-binding viruses, including adenovirus and reovirus, have been utilized for therapeutic applications such as gene-delivery vectors and oncolytic agents. Sialic acid-responsive regulatory gene circuits engineered or sialidase bio-orthogonally decorated onto bacteria have been developed for solid tumor therapy [334]. Siglec-knockout chimeric antigen receptor-macrophages showed strong anticancer activity [335]. Engineered sialylation is employed for half-life extension for glycotherapeutic proteins due to its non-clearance by asialoglycoprotein receptors (ASGPRs) in the liver [336] (Figure 5A). Sialic acid-coated liposomes [337] or lipid nanoparticles (LNPs) are utilized for tumor-specific targeted delivery (Figure 5B).

However, there are still many unanswered questions related to sialylation. A detailed understanding of the glycogenes, such as STs, responsible for sialylation in glycoproteins and glycolipids in various tissues and organs remains to be elucidated. The causal relationship between the malfunction of molecular machineries and the pathogenic development of human diseases needs further elucidation. It is still difficult to determine which glycoproteins or glycolipids are sialylated and the exact roles of sialylation in the human systems. The challenges of dissecting the roles of the gangliosides in the brain and immune systems are attractive, and efficient experimental technologies and strategies are still lacking and should be developed. Besides the sialylated glycans on the glycoproteins, sialylated glycolipid gangliosides have also emerged as a major pathophysiologic pathway for autoimmune neuropathy. Identifying new autoantibodies that are associated with a variety of clinical syndromes of autoimmune peripheral nerve diseases and understanding the mechanisms by which hyposialylation of autoantibodies and the generation of anti-sialic acid antibodies contribute to the development of autoimmune diseases are important future research directions.

## Figures and Tables

**Table 1:** Summary of the KO phenotypes of mouse STs.

Group	ST-KO	KO Phenotypes	References
(1) Immune-related	ST3Gal-I KO	Deficiency of mature cytotoxic T lymphocytes	[154,155]
(2) Metabolic-related	ST3Gal-II KO	Late-onset obesity and insulin resistance; deceased expression of calcium-binding interneurons	[159,160]
(3) Neurological related	ST3Gal-III KO	Prevent > 95% glycosphingolipid	[166]
(2) Metabolic-related	ST3Gal-IV KO	Lead to clearance of von Willebrand factor and platelets, resulting in thrombocytopenia and bleeding disorder. With ST3Gal-VI double-deficient mice, E-selectin-dependent rolling was almost completely absent.	[149,161]
(2) Metabolic-related	ST3Gal-V KO	Increased sensitivity to insulin and loss of hearing due to degeneration of the organ of Corti. Exhibiting enhanced tumor growth and angiogenesis.	[162,163,164]
(1) Immune-related	ST3Gal-VI KO	Impaired P-selectin-dependent, but not E-selectin-dependent leukocyte rolling. Neutrophil recruitment into the inflamed peritoneal cavity and lymphocyte homing to secondary lymphoid organs were impaired.	[149]
(1) Immune-related	ST6Gal-I KO	Decrease in the proliferation of B lymphocytes or IgM-producing levels. Reduced thymic cellularity in the deficient mice starting in the early thymocyte compartments.	[156,157]
(4) Miscellaneous	ST6Gal-II KO	With no efficient enzyme activity in vivo.	[170]
(4) Miscellaneous	ST6GalNAc-I KO	Reduced sialylated glycans on the ocular mucins.	[171]
N/A	ST6GalNAc-II KO	Not available.	
(1) Immune-related	ST6GalNAc-III KO	The St6galnac3: St6galnact4 double knockout (DKO) mice showed spontaneous hemorrhage of the lymph nodes. Reduction in disialyl-T antigen levels.	[158]
(1) Immune-related	ST6GalNAc-IV KO	The St6galnac3: St6galnact4 double knockout (DKO) mice showed spontaneous hemorrhage of the lymph nodes. Reduction in disialyl-T antigen levels.	[158]
(2) Metabolic-related	ST6GalNAc-V KO	Not available. Human mutations found in an Iranian family with coronary artery disease.	[165]
N/A	ST6GalNAc-VI KO	Not available.	
(3) Neurological related	ST8Sia-I KO	No apparent effect on viability or fertility, but double KO with GalNAcT displayed a sudden death phenotype and were extremely susceptible to induction of lethal seizures by sound stimulus.	[167]
(3)Neurological related	ST8Sia-II KO	Defects in the formation of neurobiological synapses.	[168]
(3)Neurological related	ST8Sia-III KO	Unsialylated several striatum-enriched GPCRs (including A_2A_R and D_1_R /D_2_R). Altered distribution of these proteins in lipid rafts. Possibly related to several basal ganglia diseases (such as schizophrenia and Parkinson’s disease).	[169]
(1) Immune-related	ST8Sia-IV KO	Defects in the formation of neurobiological synapses and LTP deficit. A 30% reduction in total thymocytes and a concomitant deficiency in the earliest thymocyte precursors.	[168,172,173]
N/A	ST8Sia-V KO	Not available.	
N/A	ST8Sia-VI KO	Not available.	

## Data Availability

Not applicable.
